# *Mycobacterium ulcerans* in the Elderly: More Severe Disease and Suboptimal Outcomes

**DOI:** 10.1371/journal.pntd.0004253

**Published:** 2015-12-02

**Authors:** Daniel P. O’Brien, N. Deborah Friedman, Raquel Cowan, James Pollard, Anthony McDonald, Peter Callan, Andrew Hughes, Eugene Athan

**Affiliations:** 1 Department of Infectious Diseases, Barwon Health, Geelong, Victoria, Australia; 2 Department of Medicine and Infectious Diseases, Royal Melbourne Hospital, University of Melbourne, Melbourne, Victoria, Australia; 3 Manson Unit, Médecins Sans Frontières, London, United Kingdom; 4 Department of Plastic Surgery, Barwon Health, Geelong, Victoria, Australia; Komfo Anokye Teaching Hospital, GHANA

## Abstract

**Background:**

The clinical presentation of *M*. *ulcerans* disease and the safety and effectiveness of treatment may differ in elderly compared with younger populations related to relative immune defficiencies, co-morbidities and drug interactions. However, elderly populations with *M*. *ulcerans* disease have not been comprehensively studied.

**Methodology/Principal Findings:**

A retrospective analysis was performed on an observational cohort of all confirmed *M*. *ulcerans* cases managed at Barwon Health from 1/1/1998-31/12/2014. The cohort included 327 patients; 131(40.0%) ≥65 years and 196(60.0%) <65 years of age. Patients ≥65 years had a shorter median duration of symptoms prior to diagnosis (p<0.01), a higher proportion with diabetes (p<0.001) and immune suppression (p<0.001), and were more likely to have lesions that were multiple (OR 4.67, 95% CI 1.78–12.31, p<0.001) and WHO category 3 (OR 4.59, 95% CI 1.98–10.59, p<0.001). Antibiotic complications occurred in 69(24.3%) treatment episodes at an increased incidence in those aged ≥65 years (OR 5.29, 95% CI 2.81–9.98, p<0.001). There were 4(1.2%) deaths, with significantly more in the age-group ≥65 years (4 compared with 0 deaths, p = 0.01). The overall treatment success rate was 92.2%. For the age-group ≥65 years there was a reduced rate of treatment success overall (OR 0.34, 95% CI 0.14–0.80, p = <0.01) and when surgery was used alone (OR 0.21, 95% CI 0.06–0.76, p<0.01). Patients ≥65 years were more likely to have a paradoxical reaction (OR 2.06, 95% CI 1.17–3.62, p = 0.01).

**Conclusions/Significance:**

Elderly patients comprise a significant proportion of *M*. *ulcerans* disease patients in Australian populations and present with more severe and advanced disease forms. Currently recommended treatments are associated with increased toxicity and reduced effectiveness in elderly populations. Increased efforts are required to diagnose *M*. *ulcerans* earlier in elderly populations, and research is urgently required to develop more effective and less toxic treatments for this age-group.

## Author Summary

*Mycobacterium ulcerans* is an infection that can affect all age-groups. It causes necrosis of skin and soft-tissue often resulting in severe outcomes and long-term disability. However, due to the majority of infections worldwide occurring in children and young adults, there is a paucity of information available in elderly patients. It is important that elderly patients are not neglected as the clinical presentation and treatment outcomes may differ significantly from younger patients related to relative immune defficiencies, co-morbidities and increased potential for drug interactions. We specifically examined patients with M. *ulcerans* disease aged ≥ 65 years and showed that they comprise a significant proportion of patients affected in Australian populations. They present with more severe and advanced disease forms, and suffer from increased toxicity and reduced effectiveness of the currently recommended treatments. Therefore, our study demonstrates that increased efforts are required to diagnose *M*. *ulcerans* disease earlier in elderly populations, and that research is urgently required to develop more effective and less toxic treatments for this age-group.

## Introduction

*Mycobacterium ulcerans* (*M*. *ulcerans*) is an infection that causes necrotizing lesions of skin and subcutaneous tissue. The majority of cases are reported from west and central Africa, but unlike Africa where the disease occurs mainly in children[1,2], in south-eastern Victoria, Australia it occurs mainly in adults with a large proportion aged > 50 years.[3] Reported rates of disease in Australian populations are up to 7 times higher in those ≥55 years of age[4]. Current *M*. *ulcerans* treatment guidelines recommend combined antibiotics for 8 weeks with surgery as an adjunctive treatment[5,6].

The clinical presentation of *M*. *ulcerans* disease (Buruli ulcer), as well as the safety and effectiveness of treatment, may differ in elderly compared with younger populations. It is known that immune function reduces with senescence, and as the immune system plays a vital role in the control of *M*. *ulcerans*[7,8], this may lead to an increase in the incidence and severity of disease as well as reduced effectiveness of treatment. There may also be altered health-seeking behaviours in older people who may find accessing healthcare more difficult or neglect skin lesions, or for whom there is a potentially increased prevalence of alternative causes of ulceration (eg venous disease) resulting in misdiagnosis. These aforementioned issues could lead to delays in diagnosis with increased disease severity. Furthermore, increased rates of co-morbidities in elderly patients may adversely affect immune function, but may also lead to increased drug interactions and the potential for increased toxicity associated with antibiotic treatment[9].

In our practice we have observed significant numbers of elderly patients developing *M*. *ulcerans* disease. Our earlier published experience has suggested that populations older than 60 years of age may have had increased prevalence of multiple *M*. *ulcerans* lesions at presentation[3], reduced rates of treatment success with surgical treatment[10], and increased rates of antibiotic related paradoxical reactions[11]. However, populations aged ≥65 years with *M*. *ulcerans* have not been comprehensively studied. We therefore undertook to describe in an Australian cohort the proportion of patients aged ≥65 years affected by *M*. *ulcerans* and compare them with younger patients with respect to their clinical presentation, and the safety and effectiveness of treatment.

## Methods

A retrospective analysis was performed on data from a prospectively collected cohort of all confirmed *M*. *ulcerans* cases managed at Barwon Health from 1/1/1998-31/12/2014. A *M*. *ulcerans* case was defined as the presence of a lesion clinically suggestive of *M*. *ulcerans* plus any of (1) a culture of *M*. *ulcerans* from the lesion, (2) a positive PCR from a swab or biopsy of the lesion, or (3) histopathology of an excised lesion showing a necrotic granulomatous ulcer with the presence of acid-fast bacilli (AFB) consistent with acute *M*. *ulcerans* infection. Lesion size was determined by measuring the extent of lesion induration with a ruler and a WHO category was assigned according to published definitions.[5] Elderly age was defined as ≥65 years in line with the accepted definition for most developed countries[12].

Drug dosages for adults included rifampicin 10 mg/kg/day (up to a maximum of 600 mg daily), ciprofloxacin 500 mg twice daily, moxifloxacin 400 mg once daily, clarithromycin 7.5 mg/kg twice daily (up to 500 mg twice daily) and ethambutol 15 mg/kg/day. A complication of medical therapy was defined as an adverse event attributed to an antibiotic that required its cessation. In cases where it was not possible to determine which antibiotic of a combination was responsible for the complication, both antibiotics were attributed with a complication. Immune suppression was defined as current treatment with immunosuppressive medication (eg. prednisolone) or active malignancy.

Treatment failure was defined as patients developing disease recurrence within 12 months of initiating treatment. Recurrence was defined as a new lesion appearing in the wound, locally, or another part of the body that met the case definition for *M*. *ulcerans* disease within 12 months of initiating treatment. Paradoxical reactions were defined by the presence of one or both of the following features: a) clinical: an initial improvement on antibiotic treatment in the clinical appearance of a *M*. *ulcerans* lesion followed by deterioration of the lesion or its surrounding tissues, or the appearance of a new lesion(s), and b) histopathology examination of excised tissue from the clinical lesion showing evidence of an intense inflammatory reaction consistent with a paradoxical reaction[11].

### Data analysis

Data was collected prospectively using Epi-info 6 (CDC, Atlanta) and analysed retrospectively using STATA 12 (StataCorp, Texas, USA). Outcome data were censored at the time of death, disease recurrence or after 12 months of follow-up from initiation of antibiotics. Categorical variables were compared using 2x2 tables and the Chi-squared test. Medians of non-parametric variables were compared using the Wilcoxon rank sum test. Odds ratios were calculated using the Mantel-Haenszel test.

### Ethics

This study was approved by the Barwon Health Human Research and Ethics Committee. All previously gathered human medical data were analysed in a de-identified fashion.

## Results

There were 327 patients treated for *M*. *ulcerans* at Barwon Health between 1/1/1998-31/12/2014 and all were included in the study. The median patient age was 58 years (IQR 35–74 years); 131 (40.0%) were ≥65 years and 196 (60.0%) were <65 years. 165 (50.5%) were male and 162 (49.5%) female.

Three hundred and eight (94.2%) patients had 1 *M*. *ulcerans* lesion, 10 (3.1%) had 2 lesions, 6 (1.8%) had 3 lesions, 1(0.3%) had 10 lesions and 2 (0.6%) had 13 lesions. 84.9% of lesions were ulcerative and 79.9% were classified as WHO category 1. The median duration of symptoms prior to diagnosis was 42 days (IQR 28–70 days). (Table 1)

**Table 1:** Baseline patient characteristics stratified by age-group.

	Overall	Age ≥65 (n = 131)	Age < 65 (n = 196)	p-value*
Sex (n = 327)				
male	165 (50.5)	56 (42.8)	109 (55.6)	0.02
female	162 (49.5)	75 (57.3)	87 (44.4)	
WHO category (n = 298)				
1	238 (79.9)	89 (74.2)	149 (83.7)	<0.01
2	33 (11.1)	11 (9.2)	22 (12.4)	
3	27 (9.1)	20 (16.7)	7 (3.9)	
Type of initial lesion (n = 325)				
Ulcer	276 (84.9)	108 (83.1)	168 (86.2)	0.18
Nodule	19 (5.9)	7 (5.4)	12 (6.2)	
Plaque	3 (1.2)	0 (0)	3 (1.5)	
Oedema	27 (8.3)	15 (11.5)	12 (6.2)	
Median duration of symptoms (n = 312) (days)	42 (IQR 28–70)	35 (21–60)	42 (28–70)	p<0.01
Number of lesions (n = 327)				
one	308 (94.2%)	118 (90.1)	190 (96.9)	<0.01
Multiple	19 (5.8%)	13 (9.9)	6 (3.1)	
Site of initial lesion (n = 327)				
Upper limb	114 (34.9)	48 (36.6)	66 (33.7)	0.08
Lower limb	203 (62.0)	80 (61.1)	127 (64.8)	
Trunk	7 (2.1)	0 (0)	3 (1.5)	
Head	3 (0.9)	3 (2.3)	0 (0.0)	
Joint	129 (39.4)	54 (41.2)	75 (38.3)	0.59
Co-morbidities				
Diabetes (n = 327)	28 (8.6)	20 (15.3)	8 (4.1)	<0.001
Immune suppression (n = 327)	28 (8.6)	22 (16.8)	6 (3.1)	<0.001

* Comparing age ≥65 to age <65 years.

### Comparison of baseline characteristics between age-groups

There were some significant differences in baseline characteristics between the age-groups. (Table 1) Patients in the age-group ≥65 years were less likely to be male (OR 0.60, 95% CI 0.38–0.94, p = 0.02), the median duration of symptoms prior to diagnosis was significantly shorter (35 compared to 42 days, p<0.01), and there was a higher proportion of patients with diabetes (p<0.001) and immune suppression (p<0.001).

### Clinical

Patients in the age-group ≥65 years were more likely to have lesions that were multiple (OR 3.49, 95% CI 1.26–9.54, p<0.01) and classified as WHO category 3 compared with category 1 and 2 combined (OR 4.89, 95% CI 1.95–12.25, p<0.001). They also had a higher proportion of oedematous compared to non-oedematous lesions (11.5% compared with 6.2%, p = 0.09). (Table 1)

### Treatment

Two hundred and eighty (85.6%) patients received antibiotic treatment for a median of 56 days (IQR 49–83 days). 115 (87.8%) of those ≥ 65 years received antibiotics and 165 (84.2%) of those < 65 years received antibiotics (p = 0.97).

There were 284 antibiotic treatment episodes in 280 patients (4 patients received a second antibiotic course—three due to disease recurrence, and 1 for a late paradoxical reaction). Initial antibiotic combinations used were rifampicin/ciprofloxacin in 162 (57.0%), rifampicin/clarithromycin in 95 (33.4%), rifampicin/moxifloxacin in 8 (2.8%), rifampicin/clarithromycin/ethambutol in 6 (2.1%), clarithromycin/ciprofloxacin in 3 (1.1%) and other varied combinations in 10 (3.5%) treatment episodes.

Overall 69 (24.3%) antibiotic treatment episodes were associated with a complication severe enough to require cessation of at least one antibiotic. There was an increased incidence of antibiotic complications in those aged ≥ 65 years compared with those aged <65 years (OR 5.29, 95% CI 2.81–9.98, p<0.001).

**Fig 1:**
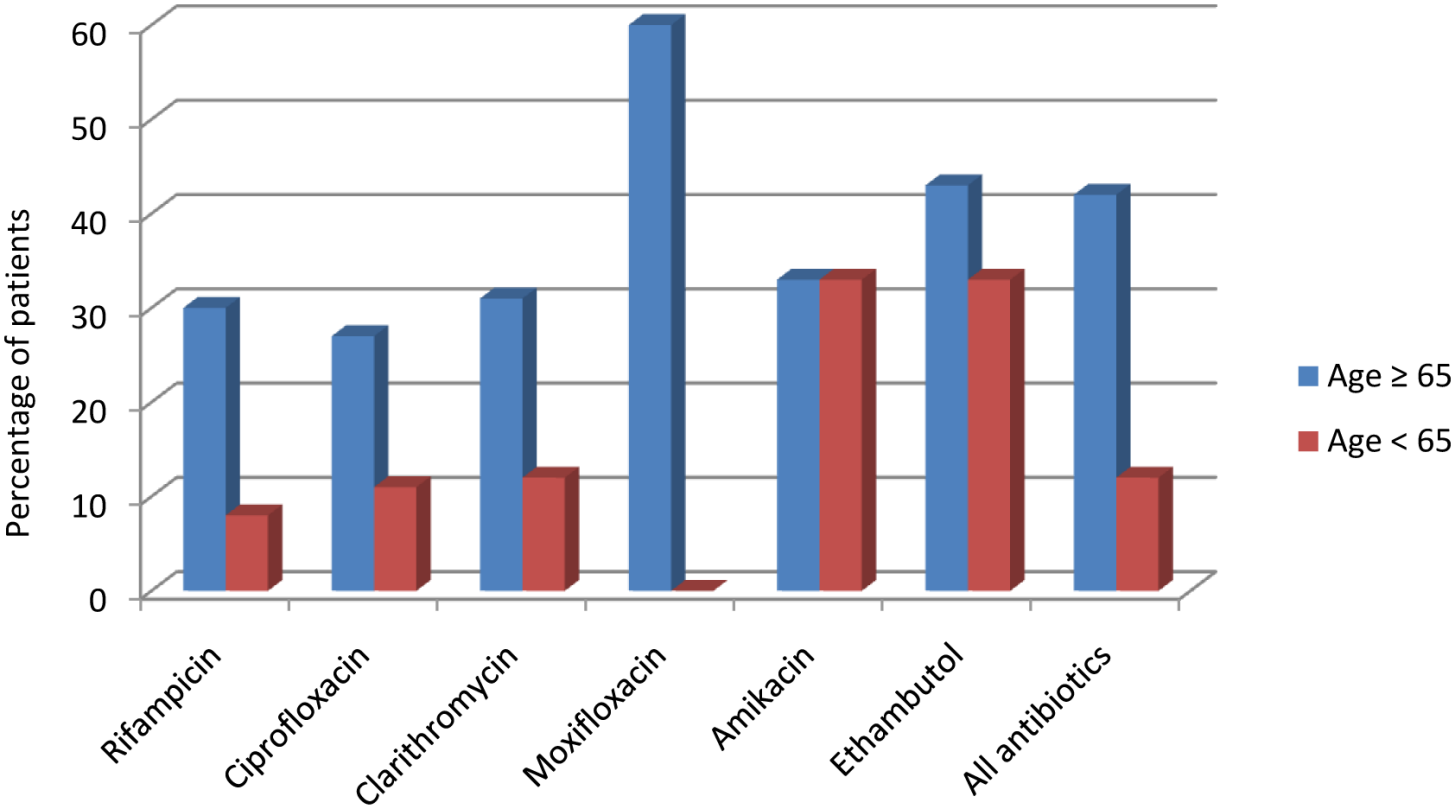
Comparison of antibiotic complications between age-groups ≥65 and <65 years.

Including antibiotics commenced as second-line treatment following cessation of one or more of the initial antibiotics due to complications, 276 (97.5%) treatment episodes included rifampicin, 174 (61.5%) ciprofloxacin, 127 (44.9%) clarithromycin, 13 (4.6%) ethambutol, 10 (3.5%) moxifloxacin and 9 (3.2%) amikacin. Rifampicin was associated with complications in 47 (17.0%) treatment episodes in which it was used, and this was more common in those aged ≥ 65 years compared to < 65 years (OR 4.87, 95% CI 2.36–10.07, p<0.001). (Table 2, Fig 1) Ciprofloxacin was associated with complications in 32 (18.4%) treatment episodes in which it was used, and this was more common in populations ≥ 65 years (OR 2.92, 95% CI 1.26–6.75, p<0.01). Clarithromycin was associated with complications in 24 (18.9%) treatment episodes in which it was used, and this was increased in populations ≥ 65 years (OR 3.38, 95% CI 1.30–8.78, p<0.01). (Table 2, Fig 1)

**Table 2:** Antibiotic complications.

Antibiotic	Overall (n,%)	Age ≥ 65 years (n,%)	Age <65 years (n,%)	p-value*	OR (95% CI)*
Rifampicin	47/276 (17.0)	34/114 (29.8)	13/162 (8.0)	<0.001	4.87 (2.36, 10.07)
Ciprofloxacin	32/174 (18.4)	22/83 (26.5)	10/91 (11.0)	<0.01	2.92 (1.26, 6.75)
Clarithromycin	24/127 (18.9)	15/49 (30.6)	9/78 (11.5)	<0.01	3.38 (1.30,8.78)
Moxifloxacin	3/10 (30.0)	3/5 (60.0)	0/5 (0.0)	<0.05	-
Amikacin	3/9 (33.0)	2/6 (33.3)	1/3 (33.3)	1.0	1.00 (0.04–22.61)
Ethambutol	5/13 (38.5)	3/7 (42.9)	2/6 (33.3)	0.74	1.50 (0.14–16.0)
All antibiotics	69/284 (24.3)	49/117 (41.9)	20/167 (12.0)	<0.001	5.29 (2.81,9.98)

* Comparing age ≥65 to age <65 years.

The specific complications associated with each antibiotic are listed in Table 3. In 11 patients hospitalization was required to manage the antibiotic complication; 9/49 (18%) in those ≥65 and 2/20 (10%) in those <65 years (OR 2.03, 95% CI 0.39–10.55, p = 0.39).

**Table 3:** Specific complications associated with individual antibiotics for all ages combined.

	Rash	GIT	Hepatitis	Renal	Blood	Tendonitis	Eye	Neurological*
Rifampicin (n = 47)	13	26	14	2	1#	0	0	0
Ciprofloxacin (n = 32)	7	19	9	2	0	6	0	0
Clarithromycin (n = 24)	6	16	5	0	0	0	0	0
Moxifloxacin (n = 3)	0	2	1	0	0	0	0	0
Ethambutol (n = 5)	0	3	0	0	0	0	2	0
Amikacin (n = 3)	0	0	0	0	0	0	0	3

GIT: gastrointestinal intolerance;

*ataxia or deafness;

^#^ thrombocytopenia

210 (64.2%) patients had surgery; More patients in the ≥ 65 years age-group had surgery compared to the < 65 years age-group [92 (70.2%) compared to 118 (60.2%), (p = 0.06)]. 62 (29.5%) had surgery alone and 148 (70.5%) had surgery plus antibiotics. There was no difference in the proportions who had surgery alone between the age-groups (p = 0.96).

### Outcomes

Treatment outcomes for first *M*. *ulcerans* lesions could be determined for 323 (98.8%) patients; 2 were lost to follow-up, 1 was transferred out, and 1 had an unclear outcome. At the time of submission, 300 (92.9%) had their outcomes determined after 12 months of follow-up and 23 (7.1%) patients after 9 months of follow-up.

There were 4 (1.2%) deaths (Table 4). The median age of those who died was 91.5 years (IQR 71.5–94.5 years), with significantly more deaths in the age-group ≥ 65 years compared to the age-group < 65 years [4 (3.1%) compared to 0 (0.0%), p = 0.01]. Only one of the deaths (#2) was felt to be directly attributable to *M*. *ulcerans* infection as a result of skin sepsis and secondary decompensated cardiac failure.

**Table 4:** Patient characteristics of those who died following commencement of MU treatment.

Patient #	Age	Sex	Year of diagnosis	Type of MU lesion	Site of MU lesion	WHO category MU lesion	Co-morbidities	MU treatment	Cause of death	Time of death after start of treatment (days)
1	94	F	2005	Ulcer	Wrist	1	Nil	52 days Rif/Cla. Surgical excision and SSG.	CVA	52
2	89	F	2008	Oedematous	Hand	3	Nil	5 days Rif/Cip. Surgical excision.	CCF, sepsis	5
3	70	F	2013	Oedematous	Leg	3	CLD, long-term prednisolone treatment	71 days Rif/Cla	Respiratory failure	186
4	95	F	2014	Oedematous	Knee	2	Carcinoma breast	67 days Rif/Cp	CVA	152

F = female; CLD = chronic lung disease; MU = Mycobacterium ulcerans; CVA = cerebrovascular accident; CCF = congestive cardiac failure; Rif = rifampicin; Cla = clarithromycin; Cp = ciprofloxacin; SSG: split skin graft

For the remaining 319 patients, the overall treatment success rate was 92.2% (Table 5); there was a reduced rate of treatment success in the age-group ≥ 65 years compared to the age-group <65 years (OR 0.34, 95% CI 0.14–0.80, p<0.01). There was a significantly reduced treatment success rate for the age-group ≥ 65 years when surgery was used alone (OR 0.21, 95% CI 0.06–0.76, p<0.01). Rates of treatment success for surgery plus antibiotics or antibiotics alone were similar between the age-groups. (Table 5)

**Table 5:** Treatment success for first MU lesions.

	All patients (n,%)	Age ≥ 65 years (n,%)	Age <65 years (n,%)	p-value*	OR (95% CI)*
All treatments	294/319 (92.2)	110/126 (87.3)	184/193 (95.3)	<0.01	0.34 (0.14, 0.80)
Surgery alone	40/61 (65.6)	12/26 (46.2)	28/35 (80.0)	p<0.01	0.21 (0.06, 0.76)
Surgery + antibiotics	144/145 (99.3)	63/64 (98.4)	81/81 (100)	0.44	0.00 (0.00–13.8)
Antibiotics alone	109/111 (98.2)	35/36 (97.2)	74/75 (98.7)	0.55	0.47 (0.01–17.91)

* Comparing age ≥65 to age <65 years.

67/275 (24.4%) patients experienced antibiotic-associated paradoxical reactions; 36 (32.4%) patients ≥ 65 years and 31 (18.9%) patients < 65 years. Patients ≥ 65 years were significantly more likely to have a paradoxical reaction compared with those < 65 years (OR 2.06, 95% CI 1.17–3.62, p = 0.01). This was independent of the WHO category of the lesion (42% v 22% for category 1, 56% v 44% for category 2 and 60% v 50% for category 3 when comparing age ≥65 to <65 years).

## Discussion

In describing a large cohort of patients aged ≥65 years, we have studied for the first time this unique population with *M*. *ulcerans* disease. Cohorts described from Africa, where most *M*. *ulcerans* cases are reported, mainly involve children with few numbers of patients aged ≥65 years[1,2]. Additionally, studies reported from Australia have focused on cohorts across all age-groups[3,13,14]. This study therefore provides important new information pertaining to the epidemiology, clinical characteristics, treatment and outcomes in elderly populations.

Patients aged ≥65 years represent an important subgroup in our cohort with *M*. *ulcerans* disease comprising two out of every 5 patients. Previous reports suggest that they may have an increased incidence of disease[4]. Additionally, our study suggests that they have more advanced and severe disease at presentation with an increased rate of multiple, large and oedematous lesions. Early non-ulcerative lesions (plaques or nodules) were infrequently reported. This is not due to late presentation as in our study the time from reported symptom onset to presentation for care was reduced in elderly patients. Instead this may be related to reduced immunity in older populations that inhibits the control of *M*. *ulcerans* leading to larger and oedematous forms of disease and the dissemination of lesions to other sites. This would be similar to the effect of HIV induced immune suppression which is associated with more severe *M*. *ulcerans* disease with an increase in the size, number and proportion of advanced lesions[15]. The reduced immunity in elderly populations may relate to the increasing immune suppression associated with senescence, and the increased presence of immunosuppressive conditions such as diabetes and malignancy or the increased likelihood of receiving immunosuppressive medication.

Our study demonstrates that treatment of *M*. *ulcerans* disease in elderly populations is associated with increased toxicity and reduced effectiveness. Nearly one-half (42%) of patients aged ≥ 65 years had to cease an antibiotic due to complications at a rate 5 times higher than younger populations, and complications were more severe with nearly one-fifth (18%) requiring hospitalization. However this cannot be avoided by treating without antibiotics as treatment with surgery alone resulted in a 79% increased failure rate in this age-group. Furthermore, there is a two-fold increase in antibiotic-associated paradoxical reactions which can cause significant morbidity and complicate treatment[11,16]. Therefore there is an urgent need to develop less toxic and more effective treatments for elderly populations.

All of the most commonly used oral antibiotics active against *M*. *ulcerans* have significant drug interactions. Rifampicin induces, and ciprofloxacin and clarithromycin inhibit, the cytochrome P450 enzyme system[17,18] leading to interactions with many commonly used medications. Clarithromycin and fluoroquinolones can prolong the QT interval creating a potential for serious arrhythmias if combined with other medical conditions or medication who do the same. This makes treatment more difficult and increases the risk of toxicity in elderly populations who are frequently prescribed multiple other medications. In addition, the pharmacokinetics of the antibiotics may differ with increasing age potentially resulting in toxic levels with currently recommended doses. For example it has been shown that elderly patients have higher serum concentrations and a longer half-life for antibiotics due to either increased bioavailability (ciprofloxacin) and reduced renal function with age (ciprofloxacin and clarithromycin) [19,20].

We advocate that pharmacokinetic and pharmacodynamic studies of frequently used antibiotics be performed in elderly patients to explore the safety and effectiveness of current and lower doses of antibiotics, including intermittent dosing regimens (e.g. thrice weekly). Further research should also be performed on the safety and effectiveness of shorter duration antibiotic regimens[21]. It would be worthwhile exploring the use of alternative antibiotics such as the new anti-tuberculous agent bedaqueline, which shows strong bactericidal activity against *M*. *ulcerans* in mouse models[22], and avermectins which have shown promising in vivo activity against *M*. *ulcerans*[23], as these may be equally effective but potentially less toxic in elderly populations.

Elderly patients were also found to have increased rates of antibiotic-associated paradoxical reactions, independent of lesion size. These likely occur due to the reversal of mycolactone induced immune suppression and the increased antigenic stimulus provided by dying mycobacteria when antibiotics are administered[24,25]. The increased rate in elderly patients in theory could relate to an increased organism load secondary to their relatively weakened immune systems which provides a greater antigenic stimulus combined with a greater potential for rapid immune function improvements when the inhibitory effects of mycolactone toxin are removed with antibiotics[11]. Increased paradoxical reactions contribute to the increased toxicity associated with antibiotics in elderly patients, and research is required to try and understand the reasons for their increased incidence in this age-group and to try and minimise their impact. Our early experience is that pre-emptive corticosteroids commenced at the initiation of antibiotics may prevent paradoxical reactions in elderly patients with oedematous lesions[26] and this should be further studied.

Finally it should be noted that *M*. *ulcerans* is not without mortality in elderly patients where sepsis secondary to skin ulceration, or complications of treatment, can contribute to death in patients with significant co-morbidities or frailty due to age.

We acknowledge the limitation that this is an observational study and as treatments were not randomized between groups there may be unmeasured confounders that may have influenced the results. However the cohort is large, data is collected prospectively and rates of follow-up are very high supporting the validity of our findings.

In conclusion, elderly patients comprise a significant proportion of *M*. *ulcerans* disease patients in Australian populations and present with more severe and advanced forms of disease. Currently recommended *M*. *ulcerans* treatments are associated with increased toxicity and reduced effectiveness in elderly populations. Increased efforts are required to diagnose *M*. *ulcerans* earlier in elderly populations, and research is urgently required to develop more effective and less toxic treatments for this age-group.
